# Global research trends related to coronavirus disease 2019 and the aged: a bibliometric analysis

**DOI:** 10.1590/1516-3180.2022.0662.R1.190523

**Published:** 2023-08-04

**Authors:** Ana Raquel Batista de Carvalho, Antonio Rosa de Sousa, Márcia Daiane Ferreira da Silva, Daniela Reis Joaquim de Freitas, Maria Eliete Batista Moura

**Affiliations:** I MSc. Doctoral Student, Postgraduate Nursing Program, Universidade Federal do Piauí (UFPI), Teresina (PI), Brazil.; II Nurse and Master's Student, Postgraduate Nursing Program, Universidade Federal do Piauí (UFPI), Teresina (PI), Brazil.; III MSc. Nurse and Professor, Universidade Estadual do Maranhão (UEMA), Coroatá (MA), Brazil.; IV PhD. Biologist, Professor, Postgraduate Nursing Program, Universidade Federal do Piauí (UFPI), Teresina (PI), Brazil.; V PhD. Nurse and Professor, Postgraduate Nursing Program, Universidade Federal do Piauí (UFPI), Teresina (PI), Brazil.

**Keywords:** COVID-19, SARS-CoV-2, Aged, Bibliometrics, Coronavirus, Pandemics, 2019-nCoV disease, Coronaviruses, Older persons, Pandemic

## Abstract

**BACKGROUND:**

A bibliometric analysis covering only the production of original studies or considering world production until 2022 has yet to be conducted. The creation and advancement of vaccines have also influenced research priorities, demonstrating the need for a new approach to this subject.

**OBJECTIVES:**

To analyze worldwide scientific production related to coronavirus disease 2019 (COVID-19) and the aged and to describe what has already been produced.

**DESIGN AND SETTING:**

Bibliometric analysis with a quantitative approach.

**METHOD:**

The search terms “COVID-19,” “SARS-CoV-2,” “Aged,” and “Elderly” were used to retrieve articles from the Web of Science database. A total of 684 articles were included in the analysis. Data were imported into RStudio Desktop Software and linked to R Software. The Bibliometrix R package and VOSviewer software were used for analysis.

**RESULTS:**

Most articles were published in 2020. These were produced by 4,937 authors and published in 379 journals. The keyword most used by the authors was “COVID-19.” Publications from 77 countries were obtained. China had the highest article production ranking, and Spain collaborated the most. The articles addressed the implications of the pandemic on the aged, the relationship between vaccination in the aged, and the implications for the disease itself.

**CONCLUSION:**

Further research should be conducted, mainly concerning vaccines and vaccination of the aged, owing to the need for and importance of immunization in this risk group, including assessing the long-term effects of vaccines.

## INTRODUCTION

On March 11, 2022, with the alarming spread and severity of coronavirus disease 2019 (COVID-19), the World Health Organization (WHO) assessed it as a pandemic. The WHO Director-General emphasized the magnitude of the new disease and the need to adopt governmental and societal approaches to create comprehensive strategies to prevent infections, save lives, and minimize the impact.^1^

With the continuation of the pandemic, based on WHO data from December 12, 2022, COVID-19 caused 645,084,824 confirmed cases worldwide, resulting in 6,633,118 deaths.^2^ This extension occurred due to the emergence of variants of the Severe Acute Respiratory Syndrome Coronavirus 2 (SARS-CoV-2) virus, such as the Omicron, Alpha, Beta, Gamma, and Delta variants. However, despite creating vaccines to prevent COVID-19, other forms of prevention are still necessary, such as using masks and hand hygiene, especially for risk groups such as older adults.^4,5^

Thus, since the beginning of the pandemic, the Centers for Disease Control and Prevention have stated that the aged are at a greater risk of developing hospital complications, extended hospital stays, and high mortality rates.^6^

Several factors require attention regarding the aged, as many belong to nursing homes or long-term institutions. For example, there is a need for strategies to prevent or impede virus transmission in these places.^7^ In addition to the occurrence of chronic diseases in this group, favoring hospitalization, health problems related to mental health can also occur, which has been affected by isolation, family distancing, or concerns about COVID-19.^8,9^

Parallel to this situation, through a bibliometric analysis of scientific literature, it is possible to identify what is being addressed in existing publications in a specific area or topic through a quantitative analysis of articles in each field so that their results can support the realization of future studies.^10^

A bibliometric analysis covering only the production of original studies or contemplating world production until 2022 is yet to be conducted.^11,12^ Additionally, the creation and advancement of vaccines have also influenced research priorities, demonstrating the need for a new approach to the subject.^5^

## OBJECTIVE

This study analyzed worldwide scientific production related to COVID-19 and the aged and described what has already been produced.

## METHODS

### Research design

This bibliometric analysis used a quantitative approach. Bibliometrics is a discipline that seeks to measure scientific and social activity and predict trends through literature analysis conducted using the following steps: research design, a compilation of bibliometric data, data analysis, data visualization, and interpretation of results.^13,14^

### Data-gathering period

A search for scientific articles was conducted using an advanced query in the Web of Science™ (WoS) database on October 31, 2022. WoS is among the most reliable and comprehensive databases for bibliometric studies, allowing the tracking of ideas across disciplines and a time of nearly 1.9 billion references cited in more than 171 million records.^15^

### Selection criteria

The included studies met the following criteria: original research on COVID-19 and the aged, without language restrictions, and published until October 31, 2022. As exclusion criteria: articles that deviated from the research scope, review articles, opinion articles, reflection articles, editorials, and case studies.

### Data-gathering

Before starting the research, the descriptors “COVID-19,” “SARS-CoV-2,” and “Aged” were obtained in the Medical Subject Headings (MeSH), including the alternative descriptor “Elderly” often used to refer to the aged in the scientific literature. To ensure precise and targeted outcomes while minimizing false positives, the present research focused exclusively on the articles using the following search strategy: “TI=((“COVID-19” OR “ SARS-CoV-2”) AND (“Aged” OR “Elderly”)).”

The search yielded 1,466 articles; 968 had early or open access after filtering the originals. To ensure the inclusion of articles on the target topic and reduce false positives, the researchers read all titles and excluded those unrelated to COVID-19 and the aged. The remaining 684 contained all available information downloaded in text file format for analysis.

### Data processing and analysis

The recovered data was imported into RStudio Desktop Software, version:2022.07.1 (^©^ Posit Software, Massachusetts, United States, 2022), linked to R Software, version:4.2.1 (The R Foundation, Vienna, Austria, 2022). For analysis, the following were used: the Bibliometrix R package (^©^ K-Synth Srl, Academic Spin-Off of the University of Naples Federico II, Naples, Italy, 2022), its graphical web interface, Biblioshiny, and VOSviewer Software, version:1.6.18 (^©^ Centre for Science and Technology Studies, Leiden University, Leiden, The Netherlands, 2022).^16,17^

In summary, the analysis allowed data visualization for later interpretation. The results encompassed various aspects, including the number of articles published annually, scientists’ productivity (Lotka's Law of 1926),^18^ the dispersion of scientific knowledge through journals (Bradford Law of 1934),^18^ the topics addressed, the most cited manuscripts, the origin of the articles, and collaborations among researchers based on their countries of origin.

## RESULTS

**Figure 1:**
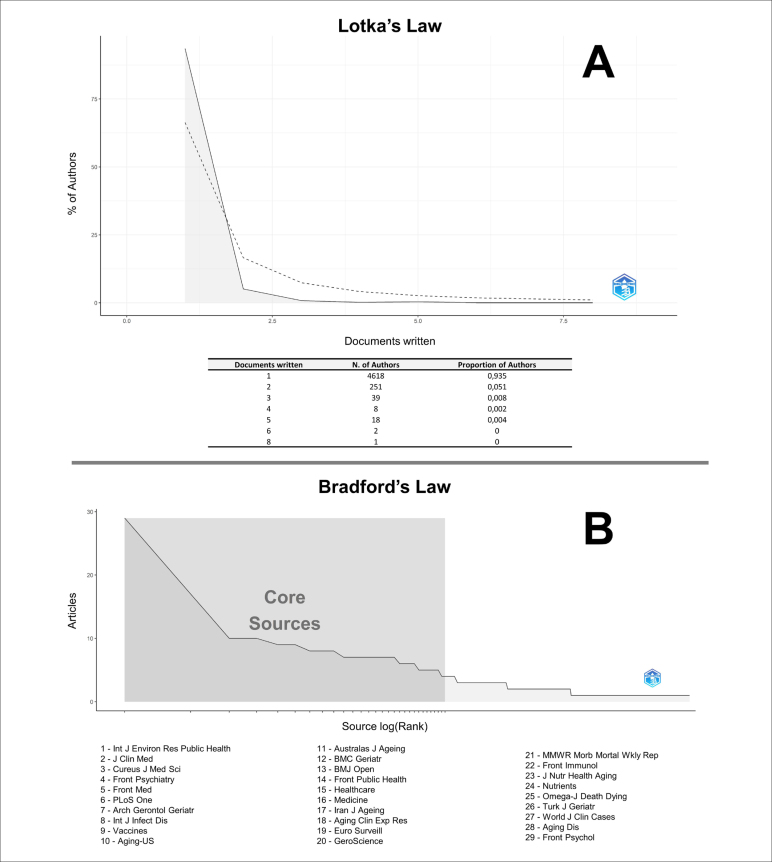
(A) Productivity of scientists according to Lotka's Law. (B) Dispersion of scientific knowledge according to Bradford's Law.

The sample comprised 684 articles, of which 17.5% (n = 120) were published in 2020, 43.9% (n = 300) in 2021, and 38.6% (n = 264) in 2022. Articles were produced by 4 937 different authors, with 93.5% of the authors present in only one article (Figure 1A). Moreover, the articles were published in 379 scientific journals, emphasizing 29 articles on the Bradford nucleus, comprising 226 articles (Figure 1B).

**Figure 2:**
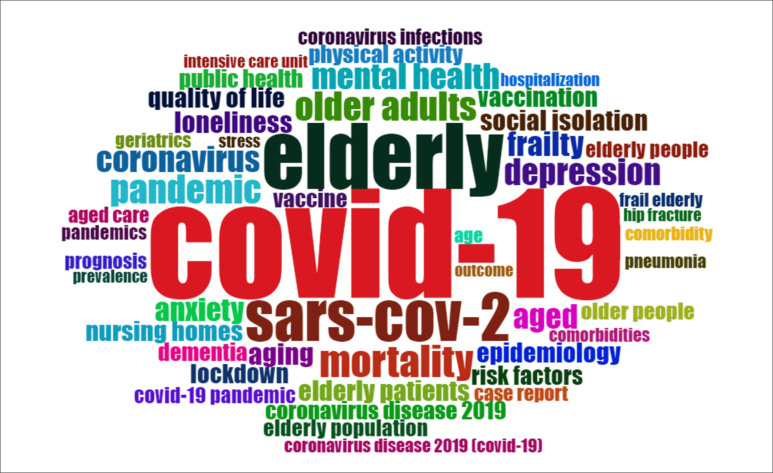
Cloud of the keywords most used by the authors.

Figure 2 shows the 49 keywords most frequently used by the authors (frequency ≥ 6). The authors used 3,212 keywords with 1,571 words. The most frequent keywords were: “COVID-19” (n = 439), “Elderly” (n = 160), and “SARS-CoV-2” (n = 86). Other words that stood out were: “mortality,” “older adults,” “pandemic,” “depression,” “coronavirus,” “mental health,” “aged,” “frailty,” and “anxiety.”

**Figure 3:**
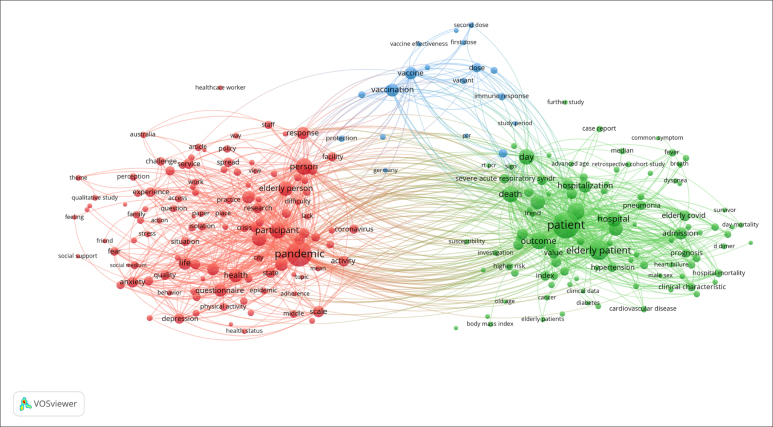
Co-occurrence of the terms of the title and the abstract.

Considering the co-occurrence of terms in frequency ≥ 10 and relating the title to the abstract, 397 terms were found from 14,170 expressions. Figure 3 shows 60% (n = 238) of the most relevant terms. The VOSviewer Software divides the terms into three main clusters, identified by the colors red, blue, and green. Noteworthy, the size of the item's circle or node is proportional to the number of times a given item appears.

The evaluated articles had an average citation count of 7.7%, using 18 434 references. In ranking the most cited articles (Table 1), the number of citations ranged from 223 to 53. Such articles have been published in 17 journals, 13 in 2020 and five in 2021.

**Table 1:** Ranking of the most cited articles on COVID-19 and the aged

Rank	Authors (year), Journal	Title	Total citations
1	Wu et al. (2021), Lancet Infect Dis^19^	Safety, tolerability, and immunogenicity of an inactivated SARS-CoV-2 vaccine (CoronaVac) in healthy adults aged 60 years and older: a randomised, double-blind, placebo-controlled, phase 1/2 clinical trial	223
2	Liu et al. (2020), Complement Ther Clin Pract^20^	Respiratory rehabilitation in elderly patients with COVID-19: A randomized controlled study	202
3	Liu et al. (2020), Eur J Clin Nutr^21^	Prevalence of malnutrition and analysis of related factors in elderly patients with COVID-19 in Wuhan, China	149
4	Tenforde et al. (2021), MMWR Morb Mortal Wkly Rep^22^	Effectiveness of Pfizer-BioNTech and Moderna vaccines against COVID-19 among hospitalized adults aged ≥65 years – United States, January-March 2021	132
5	Ioannidis et al. (2020), Environ Res^23^	Population-level COVID-19 mortality risk for non-elderly individuals overall and for non-elderly individuals without underlying diseases in pandemic epicenters	124
6	Daoust (2020), PLoS One^24^	Elderly people and responses to COVID-19 in 27 Countries	116
7	Covino et al. (2020), Geriatr Gerontol Int^25^	Clinical characteristics and prognostic factors in COVID-19 patients aged ≥80 years	86
8	Gorrochategi et al. (2020), Am J Geriatr Psychiatry^26^	Stress, anxiety, and depression in people aged over 60 in the COVID-19 outbreak in a sample collected in Northern Spain	68
9	Fulzele et al. (2020), Aging Dis^27^	COVID-19 virulence in aged patients might be impacted by the host cellular microRNAs abundance/profile	66
9	Gou et al. (2020), Gerontology^28^	Clinical characteristics of elderly patients with COVID-19 in Hunan Province, China: a multicenter, retrospective Study	66
10	Brandén et al. (2020), Lancet Healthy Longev^29^	Residential context and COVID-19 mortality among adults aged 70 years and older in Stockholm: a population-based, observational study using individual-level data	62
11	Poloni et al. (2020), EClinicalMedicine^30^	Prevalence and prognostic value of Delirium as the initial presentation of COVID-19 in the elderly with dementia: an Italian retrospective study	61
11	Moline et al. (2021), MMWR Morb Mortal Wkly Rep^31^	Effectiveness of COVID-19 vaccines in preventing hospitalization among adults aged ≥65 years – COVID-NET, 13 States, February-April 2021	61
12	Van Jaarsveld (2020), Front Psychiatry^32^	The effects of COVID-19 among the elderly population: a case for closing the digital divide	59
13	Abouhashem et al. (2020), Antioxid Redox Signal^33^	Is low alveolar type II cell SOD3 in the lungs of elderly linked to the observed severity of COVID-19?	58
14	Jang and Rhee (2020), Int J Infect Dis^34^	Three cases of treatment with nafamostat in elderly patients with COVID-19 pneumonia who need oxygen therapy	54
15	Jung et al. (2021), Crit Care^35^	The impact of frailty on survival in elderly intensive care patients with COVID-19: the COVIP study	53
15	Gallè et al. (2021), Vaccines (Brasel)^36^	Acceptance of COVID-19 vaccination in the elderly: a cross-sectional study in Southern Italy	53

**Figure 4:**
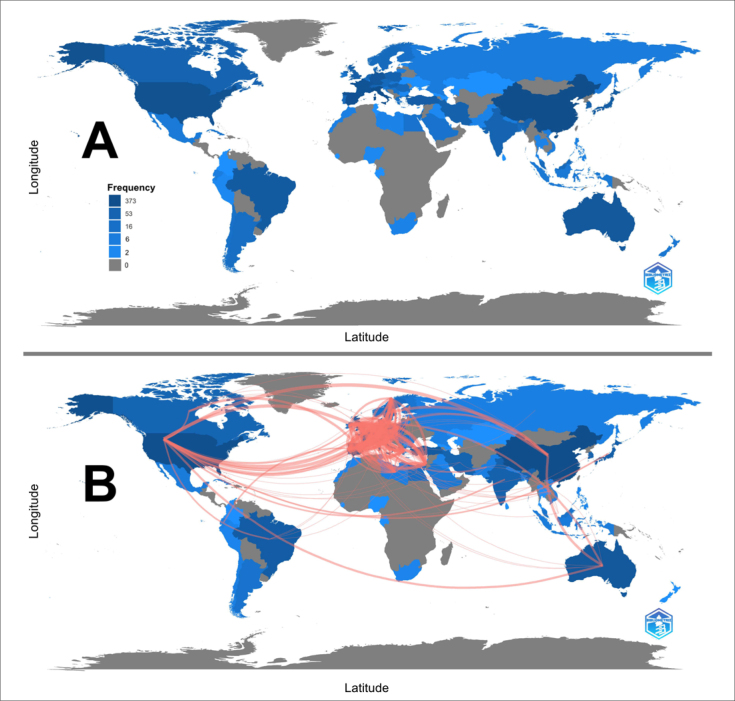
(A) (A) Co-occurrence of countries in the address list of each author. Color targeting includes gray (no posts) and blue (with posts). (B) Collaboration between producing countries. The red lines represent collaborations, with the thickness indicating the number of co-publications.

Authors from 77 countries participated in the articles, as recognized by the Bibliometrix. Figure 4A shows the countries that produced the most, considering the co-occurrence of these countries in the address list of each author, and that, consequently, may be present more than once. China occupied the top position of production by being present 373 times, followed by Italy with 329, the United States with 262, Spain with 246, and France with 204 registrations. Countries such as Germany, Australia, Brazil, and Japan also stand out.

Figure 4 B shows the collaborations, with Spain standing out for collaborating with 53 countries, France with 49 countries, and Italy with 55 countries. United Kingdom, Germany, the United States, Israel, and Poland collaborated with 51, 50, 56, 41, and 39 countries, respectively.

## DISCUSSION

This bibliometric analysis of research related to COVID-19 and the aged covered the original production from 2020 to October 2022, based on data retrieved from WoS. Regarding production, articles increased by approximately 26.4% from 2020 to 2021 and decreased by approximately 5.4% from 2021 to 2022. The increase in publications from 2020 to 2021 may have occurred because of the creation of vaccines, which began to be approved on January 5, 2021, and led to extensive scientific production related to the subject.^1,4^

Most authors have published only one article on this topic. Therefore, according to Lotka's law, this area must be consolidated. This law estimates that approximately 60% of authors will produce a single document, and a third of the literature will be produced by a tenth of the most productive authors.^18^

Moreover, the percentage of authors who produced only one article may be even more significant than what was identified. Notably, a high number of authors was noted in some articles, and there was a lack of standardization of citations of authors’ names, including the similarity of names. Such findings make it impossible to identify and make inferences about the most productive ones.

The “International Journal of Environmental Research and Public Health” (2021, Journal Citation Reports™:4,614) published the most significant number of articles on the subject (29 in total), which may be correlated with the fact that the journal is multidisciplinary, comprehensive, and composed of 20 main sections. The second journal with the most publications was the “Journal of Clinical Medicine” (2021, Journal Citation Reports™:4,964), with 17 publications; in its scope, it is defined as a comprehensive journal that accepts clinical and pre-clinical research, as well as also encourages the publication of negative results, so that other researchers do not have to repeat the experiments that other people have already performed.

The third place was occupied by two other journals, one related to psychiatry and the other related to general health, each publishing ten articles. Thus, the authors preferred to publish their studies in a broad scope because, of the scientific journals that comprised the Bradford nucleus, only 10 had a specific scope for geriatrics, gerontology, or aging.

Keywords can summarize the focus of articles and determine research trends based on the analysis of these words.^37^ In the current research, the authors’ keywords mainly addressed the name of the disease and its variations, the name of the virus, problems related to the pandemic and the disease, factors that make the aged more susceptible, and words related to vaccines or vaccination.

Several names were used to refer to the aged, such as “Elderly,” “Older adults,” “Aged,” “Aging,” “Elderly patients,” “Elderly population,” “Older people,” “Elderly people,” “Age,” “Geriatrics.” Noteworthy, such a diversity of names made it difficult to initially filter the articles, thus recommending the standardization of “Aged,” the primary term according to MeSH, or its alternative term, “Elderly.”

According to the terms of the titles and abstracts of the articles, three topics were addressed:1 (red) implications of the pandemic on the aged, which triggered psychological problems such as depression and anxiety; 2 (blue) the relation of vaccine or vaccination in the aged; and 3 (green) implications of the disease itself, causing hospitalization to death, mainly in the studied population.

Regarding the most-cited articles, despite being recent, vaccines have already received many citations. Its effectiveness was addressed in three of the most cited articles, and the fourth addressed vaccination acceptance.

The most-cited article was a randomized, double-anonymized, placebo-controlled clinical trial. The main result was vaccine tolerance in healthy adults 60 years, with live SARS-CoV-2 neutralizing antibody responses not being reduced in this population. It is noteworthy that this is the first report of an inactivated SARS-CoV-2 vaccine (CoronaVac) tested in the aged (aged ≥ 60 years).^19^

The second most cited article stated that six-week respiratory rehabilitation could improve respiratory function, quality of life, and anxiety in aged patients with COVID-19, with a slight significant improvement in depression in this public.^20^ The third study identified a high prevalence of malnutrition in aged patients with COVID-19 and concluded that nutritional support should be reinforced during treatment, especially for those with diabetes mellitus, low calf circumference, or low albumin.^21^

Other most-cited articles addressed the clinical characteristics and factors predisposing older adults to the worsening of COVID-19 and the occurrence of psychological symptoms resulting from the pandemic or the disease itself.

Based on the researchers’ origins, China ranked first in article production. This finding is due to the onset of COVID-19 transmission in this location, resulting in diverse types of research being carried out to elucidate the disease.^38,39^ Notably, China had fewer collaborations than other countries, such as Spain, demonstrating that most of its articles covered only national territory.

Another important finding relates to the nine countries that produced the most, comprising the list of the Human Development Report and classified as countries with very high or high human development. Specifically, they are countries that invest in essential universal services, such as health and education, which leads to the production of knowledge, whether by health agencies or researchers at universities.^40^

The limitations were how the bibliometric survey was conducted, including using a single database, and the rigor of the search adopted by contemplating only the titles of the articles. Another limitation was the non-standardization of authors’ names, which, owing to the similarity of names, may have influenced Lotka's law.

However, the database used is a selective, structured, and balanced database with complete citation links and improved metadata that support a wide range of information purposes, allowing the development of scientometrics.^41^ Regarding the search by title, studies have already described that it allows the recovery and specificity of the articles, generating minimal losses compared to the search that includes all fields.^42,43^

## CONCLUSION

When observing the results of the present bibliometric analysis on scientific production related to COVID-19 and the aged, it is noted that the production of new articles increased from 2020 to 2021 but has already started to decrease. Thus, the main topics addressed in the articles were the implications of the pandemic on the aged, which triggered psychological problems such as depression and anxiety; the relationship between the vaccine or vaccination in the aged; and the implications of the disease itself, which can lead to hospitalization or even death, especially in the studied population. The most-cited article, with 223 citations, addressed one of the topics already described: vaccine effectiveness (CoronaVac) in the older adult population.

Thus, synthesizing the research patterns related to COVID-19 and the international population can provide valuable insights into future research areas and perspectives. Thus, considering the current context of the COVID-19 pandemic, it is suggested that further research be conducted, mainly related to vaccines and vaccination of the aged, owing to the need for and importance of immunization in this risk group as well as the need to assess the long-term effects of vaccines.
